# Driving performance and neurocognitive skills of long‐term users of benzodiazepine anxiolytics and hypnotics

**DOI:** 10.1002/hup.2715

**Published:** 2019-12-13

**Authors:** Nick N.J.J.M. van der Sluiszen, Annemiek Vermeeren, Joris C. Verster, Aurora J.A.E. van de Loo, Joke H. van Dijken, Janet L. Veldstra, Karel A. Brookhuis, Dick de Waard, Johannes G. Ramaekers

**Affiliations:** ^1^ Department of Neuropsychology and Psychopharmacology, Faculty of Psychology and Neuroscience Maastricht University Maastricht The Netherlands; ^2^ Division Pharmacology Utrecht University Utrecht The Netherlands; ^3^ Institute for Risk Assessment Sciences Utrecht University Utrecht The Netherlands; ^4^ Centre for Human Psychopharmacology Swinburne University Melbourne Victoria Australia; ^5^ Department of Clinical and Developmental Neuropsychology University of Groningen Groningen The Netherlands

**Keywords:** anxiolytics, benzodiazepines, driving performance, hypnotics, long‐term use

## Abstract

**Objective:**

The aim of this study is to compare actual driving performance and skills related to driving of patients using benzodiazepine anxiolytics or hypnotics for at least 6 months to that of healthy controls.

**Methods:**

Participants were 44 long‐term users of benzodiazepine and benzodiazepine‐related anxiolytics (*n* = 12) and hypnotics (*n* = 32) and 65 matched healthy controls. Performance was assessed using an on‐the‐road driving test measuring standard deviation of lateral position (SDLP, in cm) and a battery of neurocognitive tasks. Performance differences between groups were compared with a blood alcohol concentration of 0.5 mg/ml to determine clinical relevance.

**Results:**

Compared with controls, SDLP was significantly increased in hypnotic users (+1.70 cm) but not in anxiolytic users (+1.48 cm). Anxiolytic and hypnotic users showed significant and clinically relevant impairment on neurocognitive task measuring executive functioning, vigilance, and reaction time. For patients using hypnotics for at least 3 years, no significant driving impairment was observed.

**Conclusion:**

Impairing effects of benzodiazepine hypnotics on driving performance may mitigate over time following longer term use (i.e. 3 years or more) although neurocognitive impairments may remain.

## 1 INTRODUCTION

Benzodiazepines are frequently prescribed to treat anxiety‐ and sleep‐related disorders (Cloos & Ferreira, 2009; Morin, Jarvis, & Lynch, 2007). It is well known that these drugs may cause psychomotor and cognitive impairments that interfere with daily activities such as operating a vehicle (Dassanayake, Michie, Carter, & Jones, 2011; Vermeeren, 2004; Verster, Veldhuijzen, & Volkerts, 2005).

Epidemiological studies showed that benzodiazepine use is associated with an increase in risk of becoming involved in a traffic accident (Dassanayake et al., 2011; Neutel, 1995; Neutel, 1998; Smink, Egberts, Lusthof, Uges, & De Gier, 2010). Crash risk was highest directly following treatment initiation but gradually decreased after prolonged exposure (Neutel, 1995). Likewise, the risk of becoming involved in a traffic accident was found to be lower for repeated users of benzodiazepines in comparison with new users (Neutel, 1998). Yet, despite indications of tolerance, crash risk was still shown to be significantly higher in drivers after 1 year of benzodiazepine usage (Hansen, Boudreau, Ebel, Grossman, & Sullivan, 2015; Hemmelgarn, Suissa, Huang, Jean‐Francois, & Pinard, 1997).

In experimental studies, the clinical relevance of benzodiazepine‐induced performance impairment is established by comparison with alcohol, given its well‐documented dose‐dependent association with crash risk (Blomberg, Peck, Moskowitz, Burns, & Fiorentino, 2009; Borkenstein, Crowther, & Shumate, 1974). Most experimental studies focussed on performance impairments observed after single doses of benzodiazepines when administered to healthy volunteers (Roth, Eklov, Drake, & Verster, 2014; Vermeeren, 2004; Vermeeren, Leufkens, & Verster, 2009; Verster, Veldhuijzen, Patat, Olivier, & Volkerts, 2006). Results showed that after a single day or night of treatment, benzodiazepines produce moderate or severe impairment of driving performance equivalent to driving under the influence of a blood alcohol concentration (BAC) of 0.5 mg/ml or more. Differences between benzodiazepines with regard to their level of impairment exist and mainly depend on dosage, time of administration, and drug half‐life (Leufkens, Lund, & Vermeeren, 2009; Vermeeren, 2004; Vermeeren et al., 2009; Verster, Veldhuijzen, & Volkerts, 2004).

Data from experimental studies on drug effects on driving and neurocognitive function have also been used to classify fitness to drive. Such classification systems (de Gier, Alvarez, Mercier‐Guyon, & Verstraete, 2009; Ravera et al., 2012) express drug‐induced impairment in BAC equivalents. Classifications that are commonly used to define drug effects on driving, in relationship to alcohol, are no/minor influence (Category 0/I, BAC < 0.5 mg/ml), moderate influence (Category II, 0.5 mg/ml ≤ BAC ≤ 0.8 mg/ml), and severe influence (Category III, BAC > 0.8 mg/ml). A limitation of existing drug categorization systems is their lack of information about the effect of long‐term drug usage on driving performance. Current classifications are mainly based on acute effects of single doses or short‐term treatment in healthy volunteers. Consequently, most benzodiazepines are put in Category III because their acute effects on performance are usually severe. Yet it is known that tolerance to benzodiazepine impairment might develop after repeated administration in healthy volunteers (Ghoneim, Mewaldt, Berie, & Hinrichs, 1981; Pomara et al., 1998) and patients (O'Hanlon, Vermeeren, Uiterwijk, Van Veggel, & Swijgman, 1995; van Laar, Volkerts, & van Willigenburg, 1992).

Nevertheless, driving performance may not completely normalize, as suggested by impairment found in a range of neuropsychological functions of long‐term benzodiazepine users (Barker, Greenwood, Jackson, & Crowe, 2004; Crowe & Stranks, 2017). The severity and relevance of such impairment with respect to patients' driving performance is not clear however. The classification of benzodiazepines in Category III, irrespective of duration of use, may be overly conservative for drivers who have been receiving long‐term treatment, limiting their mobility. As a partial solution, taking duration of use into account, current Dutch laws state that benzodiazepine users are unfit to drive when treated for less than 3 years but can request an individual driver fitness evaluation after more than 3 years of stable usage (Ministry of Infrastructure and Water Management, 2000). The criterion of 3 years seems rather arbitrary, because there is no clear scientific support for this particular cut‐off point. As far as we know, there are no published studies comparing driving performance of long‐term benzodiazepine users before and after 3 years of use.

The primary objective of the present study was to evaluate driving performance of long‐term users of benzodiazepine anxiolytics and long‐term users of hypnotics separately, as compared with that of a normative control group consisting of healthy volunteers. Only users of benzodiazepines classified as Category III were included. Long‐term usage was defined as longer than 6 months. The secondary objective was to evaluate driving performance separately for patients who had been using treatment for less than 3 years and those whose use exceeded 3 years. Driving performance was assessed by a standardized highway driving test in actual traffic and various neurocognitive tests related to driving.

## 2 METHODS

### 2.1 Design

The study was designed as a multicentre trial (Universities of Maastricht, Utrecht and Groningen, the Netherlands) comparing groups of long‐term users of benzodiazepines with healthy controls. Patients treated with benzodiazepines anxiolytics and hypnotics were analysed separately, because of the difference between these groups in time of drug intake relative to time of driving. It is known that the impairing effects of benzodiazepines on driving decrease with increased time after intake. Hypnotics are taken at bedtime, and driving occurs the next day, 8 hr or more after administration. In contrast, anxiolytics are administered during the day, and driving is likely to occur within 8 hr of administration. A combination of self‐reported indication and usual time of drug administration was used to classify a patient as user of hypnotics or anxiolytics.

To explore the potential difference in impairment before and after 3 years of use, hypnotic users were subdivided into two groups based on duration of treatment, that is, long‐term use between 6 months–3 years (LT3−) and long‐term use >3 years (LT3+). Anxiolytic users could not be divided based on treatment duration due to the low sample size of this group.

### 2.2 Participants

Patients were recruited via patient organizations, hospitals, and practitioners affiliated with UPPER (Koster, Blom, Philbert, Rump, & Bouvy, 2014) and regional advertisement. Controls were recruited via flyers and advertisement in local newspapers.

Study participants were informed about the study's goal, procedures, and potential hazards. The Medical Ethics Committee of Maastricht University and the Maastricht Academic Hospital approved the study. Furthermore, the study was conducted in agreement with the code of ethics on human experimentation established by the Declaration of Helsinki (1964), amended in Edinburgh (2000), Seoul (2008), and Fortaleza (2013). Written informed consent was obtained from each volunteer before enrolment. Volunteers received a financial compensation for their participation in the study.

#### 2.2.1 Patients

A group of 44 long‐term users of benzodiazepines or benzodiazepine‐like drugs (i.e., Z drugs) was recruited (12 users of anxiolytics and 32 users of hypnotics). All patients used category III drugs that are expected to severely affect fitness to drive. These included alprazolam, bromazepam, brotizolam, diazepam, lorazepam, lormetazepam, midazolam, nitrazepam, oxazepam, temazepam, zolpidem, or zopiclone. Initial screening was based on a medical history questionnaire that was evaluated by a clinician.

The following inclusion criteria had to be met: use of a category III benzodiazepine or benzodiazepine‐like drug over a period of at least 6 months with a frequency of at least two times a week (≈90 days/year), possession of a valid driver's license for at least 3 years, driving an average of at least 500 km/year, normal or corrected to normal vision, and body mass index between 17 and 35 kg/m^2^. Although Dutch law deems benzodiazepine users who have been treated for less than 3 years are unfit to drive, many of them drive a motor vehicle simply because they are unaware of this legal provision and because this provision is not actively enforced by the Dutch government either. Patients were excluded if they used concomitant medication classified as International Council on Alcohol, Drugs and Traffic Safety (ICADTS) Category III. Concomitant medication classified as ICADTS Category 0/I was allowed, whereas ICADTS Category II was evaluated by a clinician on individual bases. Additional exclusion criteria were alcohol use >21 glasses per week, smoking >20 cigarettes a day, and use of illegal drugs.

Before test days, patients took their anxiolytic or hypnotic medication as usual, that is, in the evening or morning before testing. Patients usual dosing regimen were established at screening and monitored by self‐report on the practice and test day.

#### 2.2.2 Controls

A group of 65 healthy volunteers was recruited with comparable age, gender distribution, and driving experience as patients. Inclusion criteria were a valid driver's license for at least 3 years, driving an average of 3,000 km/year, normal or corrected to normal vision, and a body mass index between 19 and 29 kg/m^2^. Exclusion criteria were diagnosed with a neurological disorder or sleeping disorder, alcohol use >21 glasses per week, smoking >10 cigarettes a day, and use of illegal drugs and psychoactive medication (e.g., antidepressants, benzodiazepines, antiepileptics, anticonvulsants, antihistamines, and opioids).

### 2.3 Driving test

**Figure 1:**
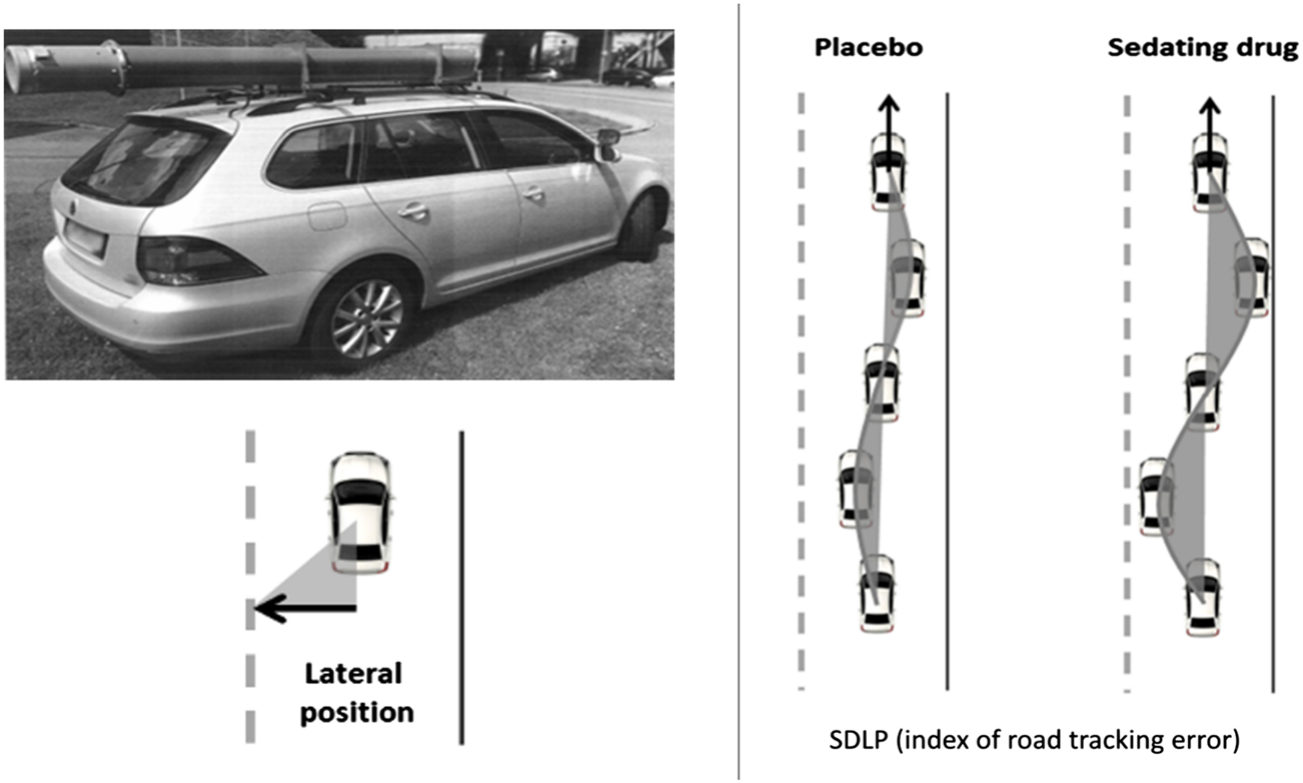
Standard highway driving test. Left: Volunteers drive a specially instrumented vehicle for about 1 hr over a 100‐km primary highway circuit, accompanied by a licensed driving instructor having access to dual controls. The volunteer's task is to drive with a steady lateral position between the delineated boundaries of the slower (right) traffic lane, while maintaining a constant speed of 95 km/hr. The lateral position of the car relative to the middle line, between the left and right traffic lane, is continuously measured by means of a camera that is mounted on the roof of the car. Right: schematic drawing of the highway driving test. The standard deviation of lateral position (SDLP) is an index of road tracking error or “weaving.” Drugs that induce sleepiness or sedation cause loss of vehicle control, leading to increased road tracking error

In the standardized on‐the‐road highway driving test (Figure 1; O'Hanlon, 1984; Ramaekers, 2017; Verster & Roth, 2011), volunteers drive a specially instrumented car over a 100 km (61 miles) primary highway circuit accompanied by a licensed driving instructor having access to dual controls. The volunteers' task is to maintain a constant speed of 95 km/hr (58 miles/hr) and a steady lateral position between the delineated boundaries of the slower right hand traffic lane. The vehicle's speed and lateral position relative to the left lane delineation is continuously recorded. These signals are digitally sampled at 4 Hz and edited offline to remove data recorded during overtaking manoeuvres or disturbances caused by roadway or traffic situations. The remaining data yield the standard deviation of lateral position (SDLP) and speed for each successive 5‐km segment and, as the square root of pooled variance over all segments, for the test as a whole. The primary outcome variable is the SDLP (in cm), which is a measure of road tracking error or “weaving.” Drug‐induced impairments in the standardized highway driving test have been compared with that of a well‐known benchmark drug (i.e., alcohol) that is known to jeopardize traffic safety and shows a clear exponential dose‐dependent relationship with accident crash risk (Blomberg et al., 2009; Borkenstein et al., 1974). The clinical relevance of performance changes in the highway driving test has previously been determined by establishing the relationship between BAC and SDLP (Louwerens, Gloerich, DeVries, Brookhuis, & O'Hanlon, 1987). A recent meta‐analysis of nine alcohol calibration studies revealed a mean increment in SDLP of 2.5 cm while operating the vehicle at a BAC of 0.5 mg/ml, which has been defined as the minimal cut‐off value to represent clinically relevant impairment (Jongen et al., 2017). The highway driving test has been used in more than 100 studies and has proven sensitivity to alcohol, benzodiazepines, and many other sedating drugs (Ramaekers, 2017; Roth et al., 2014; Vermeeren, 2004).

### 2.4 Neurocognitive tests

#### 2.4.1 Trail Making Test

The Trail Making Test (TMT) is a paper‐and‐pencil test measuring selective and divided attention, as well as executive functions (Reitan, 1958). The test comprises two parts. In Part A, the task of the volunteer is to connect, as fast as possible, 25 circles that contain the Numbers 1 to 25, by means of connecting the circles in ascending order. In Part B, the 25 circles contain letters (A to L) and numbers (1 to 13). Volunteers are required to connect, as fast as possible, the 25 circles in an alternately ascending fashion (i.e., 1‐A‐2‐B‐3‐C, and so on). The maximum time allowed for part A is 5 min, and for part B, it is 6 min. The outcome measures for Parts A and B is the time (in seconds) needed to complete the task.

#### 2.4.2 Digit Symbol Substitution Test

The Digit Symbol Substitution Test (DSST) is a paper‐and‐pencil test measuring executive attention and processing speed (Wechsler, 1958). Volunteers are presented with rows of digits (1 to 9) and have to respond by writing the corresponding symbol in a blank space, according to a key presented at the top of the paper. The primary outcome measure is the number of correctly substituted digits in 90 s.

#### 2.4.3 Adaptive Tachistoscopic Traffic Perception Test

The Adaptive Tachistoscopic Traffic Perception Test (ATTPT) assesses visual orientation ability, visual observational ability, speed of perception, and skills in obtaining a traffic overview (Schuhfried, 2009). Volunteers are presented with pictures of traffic situations for a very short duration. After each picture, volunteers are required to indicate what was in the picture, by choosing from five answer options (i.e., cars, cyclists, pedestrians, traffic signs, and/or traffic lights). Pictures are presented adaptively, meaning that the difficulty of the pictures is adapted to the abilities of the volunteer (i.e., volunteers, who perform poorly and receive pictures containing less complex traffic situations; vice versa for volunteers who perform well). The primary outcome is the number of correct answers. Time to complete the task is 10 min.

#### 2.4.4 Reaction Test

The Reaction Test (RT) assesses reaction time and motor time in response to simple and complex visual or acoustic signals (Prieler, 2008). Before the test, volunteers are instructed to lay their index finger on a pressure‐sensitive key (i.e., rest key). During the test, volunteers are required to press a target key, with their index finger, whenever a target stimulus is presented. After pressing the target key, they must return their index finger immediately to the rest key. By means of using a rest key and target key, it is possible to distinguish between reaction time (time between the presentation of the target stimulus and the moment the index finger is removed from the rest key) and motor time (the time between releasing the rest key and pressing the target key). The current experiment uses three versions of the reaction test, that is, S1, in which volunteers have to respond whenever a yellow circle is shown on screen; S2, in which volunteers have to respond whenever they hear a tone; and S3, in which volunteers have to respond whenever they see a yellow circle on screen and a hear a tone in combination; all other stimuli combinations are to be ignored. Time to complete all three versions of this task is 10 min. Outcome measures for these tests are reaction time and motor time.

#### 2.4.5 Determination Test

The Determination Test (DT) measures reactive stress tolerance, divided attention, and mental flexibility (Neuwirth & Benesch, 2007). The test measures the ability to sustain attention over a period of approximately 10 min. Volunteers are presented with visual stimuli of varying colour and sounds with a different pitch, in a serial order. For each stimulus, a predefined button has to be pressed. The presentation of stimuli is adaptive to the reaction speed of the volunteer, meaning that the interstimulus interval is shortened when volunteers make correct and fast responses and is slowed down when volunteers make mistakes or make slow responses. During the task, volunteers are presented with the following stimuli and have to press the following corresponding buttons: (a) visual coloured circles (white, yellow, red, green, and blue), each presented colour has a matching coloured key on the keyboard; (b) auditory signals (low pitch and high pitch), each auditory signal has its own response key on the keyboard; and (c) motor signals (displayed as a white rectangle on the left or right side of the bottom of the screen), each motor signal required the volunteer to press a response pad with his right or left foot, depending on the position of the white rectangle on screen. The outcome measure is the average reaction time of all responses made.

#### 2.4.6 Risk‐Taking Test Traffic

The Risk‐Taking Test Traffic (RTTT) measures risk‐taking behaviour in potentially dangerous driving situations (Hergovich, Bognar, Arendasy, & Sommer, 2005). Volunteers are presented with 24 items (i.e., video clips) that show diverse driving situations, which are described in words before they are shown on screen. Each driving situation is shown twice. During the first time, volunteers observe the entire driving situation. During the second time, volunteers are required to press a key on the keyboard, indicating the distance from the potential hazard at which the driving manoeuvre that has just been described becomes critical or dangerous (i.e., the point at which the volunteer would no longer perform the manoeuvre). The first item of the 24 items serves as a practice item. Time to complete the task is approximately 15 min. The variable “willingness to take risk in driving situations” is measured by obtaining the distance between the moment of a potential hazard, measured in hundreds of a second, and the moment the volunteer presses the key indicating that the potential hazard becomes critical or potentially dangerous. This distance is a measure of subjectively accepted level of risk. Higher scores indicate higher levels of subjectively accepted risk.

#### 2.4.7 Psychomotor Vigilance Test

The Psychomotor Vigilance Test (PVT) is based on a simple visual reaction time test (Dinges & Powell, 1985). The test measures the ability to sustain attention over a period of approximately 10 min. Volunteers are required to respond to a visual stimulus presented at a variable interval (2–10 s) by pressing a button with the dominant hand. The visual stimulus is the presentation of a counter that starts running from 0 to 60 s at 1‐ms intervals. Volunteers are required to respond to this visual counter as soon as they perceive it on screen by pressing the corresponding button. If a response is made, the counter stops, stays on screen for 500 ms as visual feedback for the volunteer, and disappears. During this period, a variable interval is presented, and afterwards, the next counter appears on screen. This cycle repeats until 100 stimuli have been presented on screen. If a response has not been made within 60 s, the clock resets and the counter restarts. Primary outcome measures are mean response speed and number of lapses (defined as responses with RT ≥ 500 ms; Basner & Dinges, 2011). Performance on the PVT has been calibrated for dose effects of alcohol and one night of sleep deprivation (Jongen, Perrier, Vuurman, Ramaekers, & Vermeeren, 2015; Jongen, Vuurman, Ramaekers, & Vermeeren, 2014).

### 2.5 Questionnaires

#### 2.5.1 Beck's Depression Inventory

The Beck Depression Inventory (BDI; Beck, Steer, & Carbin, 1988) is a 21‐item self‐report questionnaire measuring depression‐related symptomology. Answer options for each question range from 0 to 3. The obtained total score for the BDI serves as an indicator for the presence of depression‐related symptoms, ranging from 0 to 63. Higher total scores indicate the presence of more symptoms of depression.

#### 2.5.2 State–Trait Anxiety Index—Trait

The State–Trait Anxiety Index—Trait (STAI‐T; Spielberger, Gorsuch, & Lushene, 1970) is the trait dimension of the 40‐item self‐reported STAI questionnaire. The STAI‐T contains 20 questions that measure trait anxiety (i.e., how individuals feel in general). Answer options for each questions range from 1 to 4, with total scores ranging from 20 to 80. Higher total scores indicate more anxiety‐related symptoms.

#### 2.5.3 Pittsburgh Sleep Quality Index

The Pittsburgh Sleep Quality Index (PSQI; Buysse, Reynolds, Monk, Berman, & Kupfer, 1989) is a self‐report questionnaire that assesses the quality and patterns of sleep over the last month, by rating seven sleep‐related domains: subjective sleep quality, sleep latency, sleep duration, habitual sleep efficiency, sleep disturbance, use of medication, and daytime disturbance. A summary score ranging from 0 to 21 can be derived, with higher scores indicating poorer sleep quality. A summary score ≥5 indicates a poor sleeper.

#### 2.5.4 Groningen Sleep Quality Scale

The Groningen Sleep Quality Scale (GSQS; Mulder‐Hajonides van der Meulen, Wijnberg, Hollander, De Diana, & van den Hoofdakker, 1980) is a 14‐item self‐report scale that assess subjective quality of sleep during the preceding night. Summary scores range from 0 to 14, with higher scores indicating poorer sleep quality. A total score ≥6 indicates disturbed sleep.

### 2.6 Procedure

**Figure 2:**
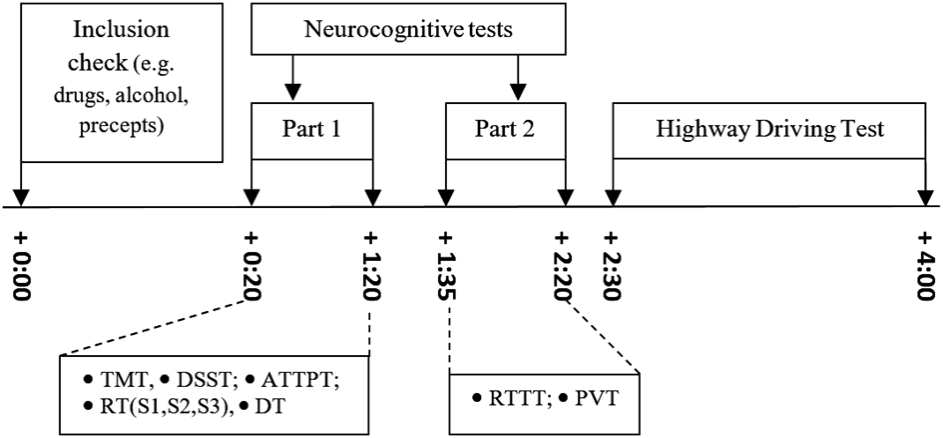
Schedule of a testing day. Time (in hours) is displayed relative from start. ATTPT, Adaptive Tachistoscopic Traffic Perception Test; DSST, Digit Symbol Substitution Test; DT, Determination Test; PVT, Psychomotor Vigilance Test; RT, Reaction Test; RTTT, Risk‐Taking Test Traffic; TMT, Trail Making Test

All volunteers completed a practice session and a test session, on two separate days with an interval of 1 week between both days. Volunteers started at 8:30 a.m., 10:30 a.m., or 12:30 p.m. based on individual convenience, but the starting time was kept constant on practice and test days. On Day 1 (practice day), volunteers filled out three questionnaires (BDI, PSQI, and STAI‐T) and were familiarized with the test procedures. Volunteers were individually trained to perform the driving test and all the neurocognitive tests. On Day 2 (test day), volunteers first completed the GSQS, followed by the first part of the neurocognitive test battery (TMT, DSST, ATTPT, RT, and DT). After a 15‐min break, volunteers completed the second part of the neurocognitive test battery (RTTT and PVT). Finally, volunteers were transported to the start of the highway to start the 1‐hr driving test. The total duration of a test day was approximately 4 hr (Figure 2).

### 2.7 Statistical analysis

Statistical power to detect a clinically relevant mean difference in SDLP of 2.5 cm between patients and controls was as follows: anxiolytic users versus controls, *β* = .58; hypnotic users versus controls, *β* = .85; hypnotic LT3− users versus controls, *β* = .50; and hypnotic LT3+ users versus controls, *β* = .78. Assumptions for these power calculations were an alpha of .05 and a between‐subject variance in SDLP of 4.3 cm (Jongen et al., 2017).

Matching variables (age, gender, and driving experience) were included as covariates in an analysis of covariance model. If none of the matching variables showed a significant influence on group comparisons with SDLP or a neurocognitive parameter, patient performance was compared with that of the entire group of controls (*n* = 65). Alternatively, if one (or more) matched variable did show a significant influence on a between‐group comparison, only individually matched controls were included. The determination of the influence of matching variables was performed for SDLP and each neurocognitive parameter separately.

**Figure 3:**
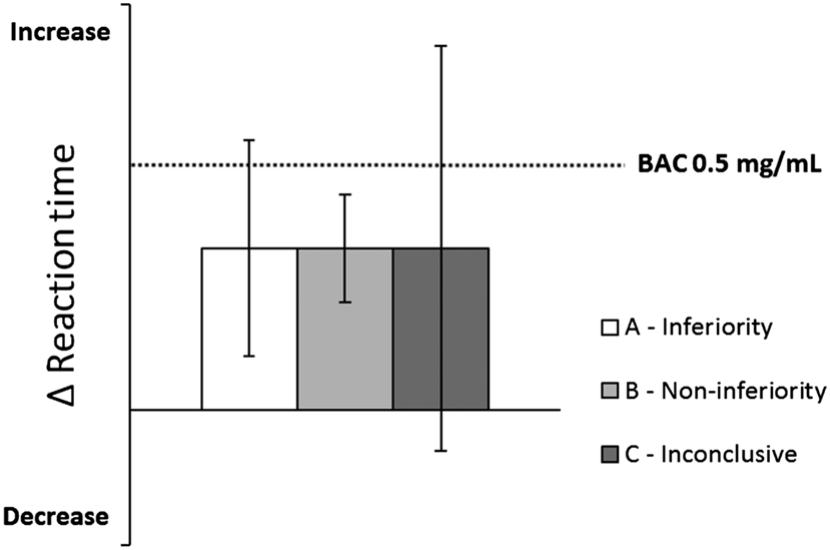
Hypothetical example of the qualification of clinical relevance of performance differences between patients and controls. The dotted line indicates the change in performance after alcohol intake (relative to placebo). A (drug induced) change in performance will be classified as inferior when the 95% confidence interval (CI) includes the alcohol criterion but not zero (A—inferiority). Noninferiority is concluded when the 95% CI does not include the alcohol criterion (B—noninferiority). If the 95% CI includes the alcohol criterion as well as zero, the qualification of clinical relevance is undecided (C—inconclusive). BAC, blood alcohol concentration

**Figure 4:**
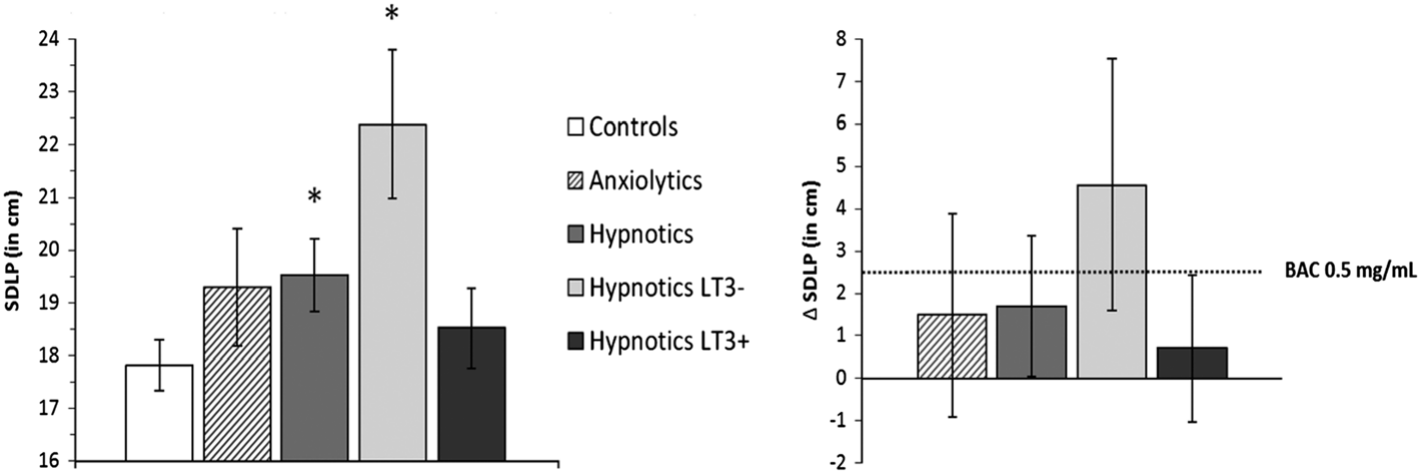
Left: mean (±standard error) standard deviation of lateral position (SDLP) for controls and patients groups. Right: mean (95% confidence interval) differences in SDLP between patient groups and controls. The dotted line indicates the change in performance after alcohol intake (relative to placebo). Symbols above bars indicate significant difference from controls, *p* < .05. BAC, blood alcohol concentration

Next, noninferiority analyses were used to determine whether the 95% confidence interval (CI) of performance differences between patients and controls exceeded the criterion level of clinical relevance, that is, an equivalent performance change as seen at a BAC of 0.5 mg/ml. When evaluating the 95% CI of differences between groups, three interpretations are possible (Figure 3). Patients' performance was considered not impaired (i.e., noninferior) when the upper limit of the 95% CI of the difference from controls was below the alcohol criterion for impairment. Patients' performance was considered impaired (i.e., inferior) when the lower limit of the 95% CI of the difference from controls was above zero and the upper limit exceeded the alcohol criterion for impairment. When the 95% CI of the difference from controls included both zero and the alcohol criterion for impairment, the results were considered inconclusive. The noninferiority limit for the on‐the‐road driving test (Figure 4) was obtained from Jongen et al. (2017).

Clinical relevance of impairment of neurocognitive performance was also based on direct comparison impairing the effects of alcohol at a BAC of 0.5 mg/ml. In a separate study (Verster et al., 2016), an alcohol calibration was performed to determine which neurocognitive parameters were able to detect impairment at a BAC of 0.5 mg/ml. Results of the calibration study showed that the only parameters sensitive for the impairing effects of alcohol were TMT‐A, DSST, RT‐S1, RT‐S2, RT‐S3, DT, and PVT. Consequently, these are the only parameters that provided noninferiority limits for the present study. The clinical relevance of neurocognitive tests used in the present study will only be discussed for these parameters.

All statistical analyses were conducted by using the IBM Statistical Package for the Social Sciences for Windows (Version 24.0.01., IBM Corp., Armonk, NY, USA). Power calculations were performed using G*Power Version 3.1 (Faul, Erdfelder, Lang, & Buchner, 2007).

## 3 RESULTS

### 3.1 Group characteristics

Table 1 summarizes the characteristics of the patient groups and control group. Age, gender, and driving experience did not differ significantly between groups. As expected, patients had on average more complaints of anxiety, depression, and sleep problems compared with controls. Differences in scores on BDI, STAI‐T, PSQI, and GSQS were significant for anxiolytic and hypnotic users, as well as for the hypnotic LT3− and LT3+ subgroups (all *p*s < .01).

**Table 1:** . Demographic data of patient and control groups

Parameters	Anxiolytic users (*n* = 12)	Hypnotic users (*n* = 32)	Hypnotics LT3− (*n* = 9)	Hypnotics LT3+ (*n* = 23)	Controls (*n* = 65)
Gender (male/female)	6/6	12/20	2/7	10/13	37/28
Age (years)	55.2 ± 9.6	55.6 ± 12.3	47.8 ± 13.5	58.7 ± 10.6	57.9 ± 10.5
Driving experience (km/year)	11,450 ± 9,050	11,777 ± 9,039	9,594 ± 4,931	12,630 ± 10,177	13,659 ± 9,477
Depression symptoms (BDI)	15.4 ± 12.5	9.2 ± 7.6	11.8 ± 8.4	8.2 ± 7.2	2.5 ± 2.8
Anxiety symptoms (STAI‐T)	48.1 ± 11.6	40.4 ± 11.8	44.1 ± 14.4	39.0 ± 10.6	27.1 ± 5.2
Sleep problems (PSQI)	6.6 ± 3.4	10.0 **±** 5.4	9.4 ± 6.2	10.2 ± 5.1	2.8 ± 2.4
Sleep complaints pretesting (GSQS)	4.4 ± 4.6	5.5 ± 4.6	5.3 ± 1.7	5.6 ± 1.0	1.4 ± 1.8

Abbreviations: BDI, Beck's Depression Inventory; GSQS, Groningen Sleep Quality Scale; LT3−, patients treated less than 3 years; LT3+, patients treated longer than 3 years; PSQI, Pittsburgh Sleep Quality Index; STAI‐T, State–Trait Anxiety Index—Trait.

Table 2 gives an overview of psychoactive medication used per patient group. All participants took their medication at least 4 days/week. Users of anxiolytics indicated they used their medication daily, and users of hypnotics reported medication use at least four nights per week. In total, 30 patients used psychoactive comedication (Table 2), mostly second‐generation antidepressants (*n* = 22) and second‐generation antipsychotics (*n* = 6). Most antidepressants were selective serotonin reuptake inhibitors and serotonin–norepinephrine reuptake inhibitors, which have minor effects on driving (Category I). Second‐generation antipsychotics can have moderate effects on driving (Category II). The proportion of patients using category II comedication was higher in the LT3− group (44%, four out of nine) than in the LT3+ group (26%, six out of 23) and the anxiolytic group (25%, three out of 12).

**Table 2:** . Psychoactive medication used per group (reported dosages in mg)

Group			Comedication					
Benzodiazepine	*M* ± *SD*	*N*	Category I	*M* ± *SD*	*N*	Category II	*M* ± *SD*	*N*
**Anxiolytic users (*n* = 12)**								
Alprazolam, b.i.d.	1.0 ± 0.7	2	(Es)citalopram	13.3 ± 5.8	3	Clomipramine	50.0	1
Bromazepam, q.d.	3.0	2	Fluoxetine	10.0	1	Olanzapine	10.0	1
Lorazepam, t.i.d.	0.75 ± 0.4	2	Paroxetine	23.0 ± 24.0	2	Oxycodone	45.0	1
Oxazepam, b.i.d./t.i.d.	10.4 ± 2.5	6	Venlafaxine	119 ± 8.8	2	Quetiapine	150.0	1
**Hypnotic users, LT3− (*n* = 9)**								
Lorazepam, nocte	1.0	2	(Es)citalopram	30.0 ± 11.5	4	Duloxetine	60.0	1
Lormetazepam, nocte	2.0	1	Fluoxetine	30.0	1	Lithium	900.0 ± 141.4	2
Midazolam, nocte	3.75	1	Sertraline	100.0	1	Quetiapine	50.0	1
Temazepam, nocte	20.0 ± 8.2	4				Risperidone	2.0	1
Zolpidem, nocte	10.0	1						
**Hypnotic users, LT3+ (*n* = 23)**								
Brotizolam, nocte	0.25	1	Cetirizine	10.0	1	Duloxetine	30.0	1
Diazepam, nocte	5.0	1	Citalopram	17.5 ± 5	4	Lamotrigine	10.0	1
Lorazepam, nocte	1.1 ± 0.1	3	Fluoxetine	40.0	1	Oxycodone	10.0	1
Lormetazepam, nocte	3.0	1	Methylphenidate	30.0	1	Pramipexol	0.35	1
Midazolam, nocte	15.0	1	Paroxetine	20.0	3	Pregabalin	75.0	1
Nitrazepam, nocte	4.4 ± 0.9	2				Quetiapine	237.5 ± 300.5	2
Oxazepam, nocte	17.5 ± 9.6	4				Tranylcypromine	80.0	1
Temazepam, nocte	15.0 ± 7.1	2						
Zolpidem, nocte	11.7 ± 2.9	3						
Zopiclone, nocte	7.5	5						

Abbreviations: b.i.d., twice a day; LT3−, patients treated less than 3 years; LT3+, patients treated longer than 3 years; nocte, bedtime administration; q.d., once a day; t.i.d., three times a day.

### 3.2 Missing data

Data from the highway driving test were missing for one person in the control group, and for one patient in the hypnotic LT3− subgroup, due to problems with the recording system.

### 3.3 Matching to controls

Analyses showed no significant effect of age, gender, or driving experience in the analysis of covariance model on SDLP, ATTPT, RTTT, and PVT mean reaction time. For these parameters, the entire control group sample was used as a reference for comparison with patient groups. For the remaining parameters, matched healthy controls were used for each patient (sub)group.

### 3.4 Highway driving test

Mean (±standard error) SDLP of patient (sub)groups and controls is shown in Figure 4. Mean SDLP of patients using hypnotics differed significantly from controls, *F*(1, 93) = 4.12, *p* = .04. The upper limit of the 95% CI of this difference (+1.70 cm, 95% CI [+0.04 cm, +3.35 cm]) exceeded the +2.5‐cm criterion, indicating clinically relevant impairment. The SDLP of patients using anxiolytics did not significantly differ from controls, *F*(1, 74) = 1.50, *p* = .23. The 95% CI of the mean difference (+1.48 cm, 95% CI [−0.93 cm, +3.88 cm]) included zero as well as the +2.5‐cm criterion, which indicates that the results are inconclusive.

Analysis of variance (ANOVA) also showed a significant difference between LT3− hypnotic users and controls, *F*(1, 71) = 9.38, *p* < .01, but not between LT3+ hypnotic users and controls, *F*(1, 85) = 0.64, *p* = .43. Mean (95% CI) difference in SDLP between LT3− patients and controls was +4.56 cm (+1.59 cm, +7.53 cm) and +0.70 cm (−1.04 cm, +2.44 cm) for LT3+ patients. Noninferiority testing revealed that only for LT3− patient the lower and upper limit of the mean difference in overall SDLP exceeded zero and the +2.5‐cm criterion, respectively, indicating clinically relevant impairment.

### 3.5 Neurocognitive performance

Table 3 shows the mean (±standard error) for all performance parameters for each patient (sub)group and healthy controls and the results from ANOVA analyses. Table 4 shows an overview of the 95% CI of mean changes between patients and (matched) controls on alcohol‐sensitive parameters only, including inferiority limits and analyses. Comparisons between patients using anxiolytics and controls showed significant impairment of patients' performance on the DT and PVT_MeanRT_. The 95% CI of mean changes in reaction time in the DT and PVT was above zero and exceeded the BAC of 0.5‐mg/ml criterion, indicating clinically relevant impairment.

**Table 3:** . Mean (±standard error) of all performance parameters for each patient group and the (matched) healthy control group and results from analysis of variance

All controls	Anxiolytics	Hypnotics	Hypnotics LT3−	Hypnotics LT3+	All controls
	*N* = 12	*N* = 32	*N* = 9	*N* = 23	*N* = 65
SDLP (cm)	19.3 ± 1.1	19.5 ± 0.7*	22.4 ± 1.4*	18.5 ± 0.8	17.8 ± 0.5
ATTPT (number correct)	94.1 ± 3.6	96.0 ± 2.1	96.7 ± 4.1	95.8 ± 2.5	98.0 ± 1.5
RTTT	7.9 ± 0.4	7.1 ± 0.3*	8.3 ± 0.5	6.6 ± 0.3*	7.9 ± 0.2
PVT mean RT (ms)	328 ± 1*	311 ± 7*	320 ± 14*	309 ± 7*	289 ± 5

Matched controls	Anxiolytics	Matched controls	Hypnotics	Matched controls	Hypnotics LT3−	Matched controls	Hypnotics LT3+	Matched controls
	*N* = 12	*N* = 11	*N* = 32	*N* = 24	*N* = 9	*N* = 6	*N* = 23	*N* = 18
TMT‐A (s)	34.5 ± 3.3	31.5 ± 3.5	35.2 ± 2.1	29.9 ± 2.4	29.3 ± 2.6	30.3 ± 3.2	37.5 ± 2.7	29.8 ± 3.0
TMT‐B (s)	72.7 ± 9.5	61.7 ± 10.0	77.3 ± 5.6*	59.4 ± 6.3	65.0 ± 11.1	51.1 ± 13.6	82.4 ± 6.4*	62.1 ± 7.1
DSST (number correct)	52.3 ± 3.1	57.0 ± 3.2	53.5 ± 1.8*	62.0 ± 2.0	53.6 ± 3.4*	67.7 ± 4.1	53.4 ± 2.0*	60.2 ± 2.3
RT‐S1								
Reaction time (ms)	280 ± 112	273 ± 12	303 ± 10*	265 ± 12	331 ± 28	270 ± 34	291 ± 10	263 ± 10
Motor time (ms)	170 ± 18	151 ± 8	199 ± 12*	151 ± 9	213 ± 21*	151 ± 8	193 ± 14	151 ± 8
RT‐S2								
Reaction time (ms)	217 ± 9	223 ± 10	257 ± 10*	225 ± 12	285 ± 20*	213 ± 25	246 ± 11	230 ± 13
Motor time (ms)	150 ± 12	148 ± 12	193 ± 11*	146 ± 13	217 ± 24*	124 ± 29	184 ± 13*	153 ± 15
RT‐S3							
Reaction time (ms)	431 ± 20	437 ± 12	490 ± 17*	429 ± 19	534 ± 32*	418 ± 39	473 ± 19	433 ± 22
Motor time (ms)	181 ± 20	179 ± 20	229 ± 13*	167 ± 15	243 ± 31	142 ± 39	224 ± 13*	176 ± 15
DT reaction time (ms)	963 ± 34*	836 ± 36	942 ± 21*	833 ± 25	932 ± 41*	787 ± 50	946 ± 25*	848 ± 28
PVT Lapses (number)	3.2 ± 0.7	1.4 ± 0.7	3.3 ± 0.8	1.8 ± 0.9	4.9 ± 2.8	1.0 ± 3.2	2.7 ± 0.6	2.1 ± 0.7

Abbreviations: ATTPT, Adaptive Tachistoscopic Traffic Perception Test; DSST, Digit Symbol Substitution Test; DT, Determination Test; LT3−, patients treated less than 3 years; LT3+, patients treated longer than 3 years; PVT, Psychomotor Vigilance Test; RT, Reaction Test; RTTT, Risk‐Taking Test Traffic; SDLP, standard deviation of lateral position; TMT, Trail Making Test.

* Indicates significant difference from (matched) control group.

**Table 4:** . Mean (95% confidence interval) differences in alcohol calibrated neurocognitive parameters between patient and control groups and noninferiority analysis

Parameters	Noninferiority limit	Anxiolytics *N* = 12		Hypnotics *N* = 32		Hypnotics LT3− *N* = 9		Hypnotics LT3+ *N* = 23	
TMT‐A (s)	+2.72	+2.9 (−7.1, +12.9)	Inc	+5.3 (−1.2, +11.7)	Inc	−0.9 (−9.9, +8.0)	Inc	+7.7 (−0.5, +15.9)	Inc
DSST (number correct)	−1.38	+13.8 (−17.6, +39.7)	Inc	−8.6 (−13.9, −3.2)	Inf	−14.1 (−25.7, −2.6)	Inf	−6.7 (−12.9, −0.5)	Inf
RT‐S1									
Reaction time (ms)	+10.35	+6 (−28, +41)	Inc	+38 (+6, +70)	Inf	+62 (−34, +157)	Inc	+28 (−1, +58)	Inc
RT‐S2									
Reaction time (ms)	+7.83	−6 (−35, +22)	Inc	+32 (+1, +62)	Inf	+72 (+2, +141)	Inf	+17 (−18,+51)	Inc
RT‐S3									
Reaction time (ms)	+23.82	−7 (−68, +55)	Inc	+62 (+11, +112)	Inf	+116 (+8, +224)	Inf	+40 (−18, +99)	Inc
DT									
Reaction time (ms)	−9.14	+127 (+25, +229)	Inf	+109 (+44, +175)	Inf	+146 (+5, +286)	Inf	+98 (+21, +175)	Inf
PVT									
Mean RT (ms)	+19.36	+39 (+15, +64)	Inf	+22 (+6, +39)	Inf	+31 (+2, +60)	Inf	+20 (+4, +35)	Inf
Lapses (number)	+1.71	+1.8 (−0.3, +3.9)	Inc	+1.4 (−1.1, +3.9)	Inc	+3.9 (−5.3, +13.1)	Inc	+0.6 (−1.3, +2.4)	Inc

Abbreviations: DSST, Digit Symbol Substitution Test; DT, Determination Test; Inc, inconclusive; Inf, inferiority; LT3−, patients treated less than 3 years; LT3+, patients treated longer than 3 years; PVT, Psychomotor Vigilance Test; RT, Reaction Test; TMT, Trail Making Test.

ANOVA showed significant performance differences between patients using hypnotics and controls in the TMT‐B, DSST, RT‐S1 (motor and reaction time), RT‐S2 (motor and reaction time), RT‐S3 (motor and reaction time), DT, RTTT, and mean reaction time in PVT. Noninferiority analysis of alcohol‐sensitive parameters showed that the 95% CIs of differences in DSST, RT‐S1, RT‐S2, RTS3, and PVT exceeded zero and the alcohol criterion indicating that impairment on these parameters can be considered clinically relevant.

ANOVA showed significant performance differences between LT3− hypnotic users and controls in the DSST, RT‐S1 (motor time), RT‐S2 (motor and reaction time), RT‐S3 (reaction time), DT, and PVT mean reaction time. Noninferiority analysis showed that the 95% CIs of differences in DSST, RT‐S2, RT‐S3, DT, and PVT exceeded zero and the alcohol criterion indicating clinically relevant impairment.

ANOVA revealed significant performance differences between LT3+ hypnotic users and controls in the TMT‐B, DSST, RT‐S1 (motor time), RT‐S3 (motor time), DT, RTTT, and PVT mean reaction time. Noninferiority analysis showed that the 95% CIs of differences in DSST, DT, and PVT exceeded zero and the alcohol criterion indicating impairment.

## 4 DISCUSSION

This study aimed to compare driving performance of long‐term users of benzodiazepine or benzodiazepine‐related anxiolytics and hypnotics to that of a normative control group consisting of healthy volunteers, in order to evaluate whether classification of these drugs in Category III may be too conservative for patients who receive long‐term treatment. Overall, mean SDLP was significantly higher in patients treated with hypnotics as compared with controls, indicating their driving performance is worse than normal. This seemed mainly due to patients who had been using hypnotic less than 3 years, as the difference in SDLP form controls was significant in this group but not in those who had received hypnotics treatment for more than 3 years. Mean SDLP did not differ significantly between patients treated with anxiolytics and controls, which may be explained by a lack of power due to the small sample size and large individual variation. Both patient groups (users of hypnotics and anxiolytics) displayed increased reaction times in a number of neurocognitive tasks. In line with findings for SDLP in hypnotic users, these impairments were most prominent in patients who used these drugs for less than 3 years. Clinical relevance seemed less present in patients using hypnotics for more than 3 years.

The clinical relevance of the effects of long‐term benzodiazepines use on driving ability and neurocognitive performance was determined by comparing the average difference in performance between patients and controls with the change in performance in subjects who were under the influence of a BAC of 0.5 mg/ml, the legal limit for driving under the influence of alcohol in many countries. Previous studies employing the on‐the‐road highway driving test have demonstrated an average increase in mean SDLP of 2.5 cm in drivers operating with a BAC level of 0.5 mg/ml (Jongen et al., 2017). In the present study, the mean increase in SDLP in patients using anxiolytic or hypnotic drugs was 1.48 and 1.70 cm, respectively, relative to healthy controls. The 95% CI of these mean differences included the alcohol criterion in both groups, as well as zero in case of the anxiolytic users. As mentioned above, driving performance of anxiolytic users was inconsistent. Some individuals showed marked increments in SDLP, whereas others did not. Overall, no conclusion can be drawn for this group from these data.

Driving impairment observed in hypnotic users is of clinical relevance, because it exceeds the level of impairment associated with the legal limit of alcohol in traffic. This is in line with results from epidemiological studies showing increased risk of traffic accidents associated with the use of hypnotics (Gustavsen et al., 2008; Hansen et al., 2015; Orriols et al., 2011). Interestingly, however, severe impairment was present only in patients who used hypnotics less than 3 years, whereas no relevant impairment, as measured by SDLP, was found in those who had been using hypnotics longer than 3 years. The mean difference in SDLP between controls and hypnotic LT3− users was +4.56 cm, which is equivalent to a BAC > 0.8 mg/ml (Louwerens et al., 1987). For LT3+ users, the mean difference was only 0.70 cm, and the 95% CI remained below the alcohol criterion, indicating no relevant impairment of driving. The latter finding is in line with that from a previous driving study in insomnia patients who frequently used hypnotics (Leufkens, Ramaekers, de Weerd, Riedel, & Vermeeren, 2014). Duration of use in these patients was on average 7.7 years, and their SDLP did not differ from those of a group of normal sleepers. The difference in impairment between the LT3− and LT3+ groups in our study corresponds with gradually decreasing accident risk found in epidemiological studies following benzodiazepine treatment (Neutel, 1995; Verster et al., 2005). Although development of physiological tolerance may explain the mitigation in impairment, other factors may also play a role, such as improvements in underlying conditions, reduction in comedication, and behavioural tolerance (i.e., learning to minimize unwanted drug effects on performance by cognitive or behavioural adaptations).

Users of hypnotics and anxiolytics also demonstrated increments of reaction times as compared with controls on a number of neurocognitive tasks that exceeded the alcohol criterion of clinical relevance. In line with the results of the driving test, results of cognitive tests were mostly inconclusive for users of anxiolytics and showed clinically relevant impairment in users of hypnotics. Similar to driving impairment, psychomotor impairment in hypnotic users was most severe in patients who had been using these drugs less than 3 years.

Contrary to the absence of driving impairment, patients who used hypnotics longer than 3 years showed relevant impairment in some tests (i.e., the PVT, DSST, and DT). This finding seems in line with recent reviews (Crowe & Stranks, 2017; van der Sluiszen, Vermeeren, Jongen, Vinckenbosch, & Ramaekers, 2017) concluding that long‐term treatment with benzodiazepines can be associated with deleterious neuropsychological effects. Impairment was even found to persist following benzodiazepine withdrawal (Crowe & Stranks, 2017). The question remains what the consequences of this impairment are for driving. From the alcohol calibration of our tests, we can conclude that the impairment found in some neurocognitive tests after 3 years of hypnotic use is comparable with, or larger than, the effects found for alcohol at a BAC of 0.5 mg/ml. It can be concluded that impairment may be moderate, but the evidence that patients who have been using hypnotics more than 3 years are severely impaired is weak. So the current classification, and the associated general prohibition to drive when using these drugs, may be too strict for this group of patients. For shorter use of hypnotics, our data support the current classification as Category III. For anxiolytics, there is no evidence to support a change in the current classification.

Several limitations may be present in this study. First, there may be a selection bias, in so far as probably only patients who estimated themselves as fit to drive volunteered for the study. However, these patients may be representative for the target population, that is, long‐term users who are active car drivers. Patients who do not feel fit to drive are less likely to drive in real life. Second, it should be noted that the statistical power to detect clinically relevant impairment was much less in anxiolytic users (*n* = 12) and hypnotic LT3− users (*n* = 9), due to the low sample size in both groups. Yet differences from controls were relatively large in the LT3− group and therefore still achieved statistical significance. Third, the anxiolytic and hypnotic users in the present study formed a heterogeneous sample due to the diversity in benzodiazepines, daily dosages, time since last dosage, and comedication. Such factors may generate variability in performance between patients and hinder the interpretation of underlying mechanisms. However, it does reflect the diversity in the population of long‐term users of benzodiazepines who drive a car. Fourth, subdivision of benzodiazepine use below and above 3 years was a legislative measure as adopted in the Netherlands. Strict conclusions based on this subdivision should therefore be avoided. Nonetheless, future studies could explore the time needed to build op tolerance to the impairing effects of daily antidepressant usage and handle treatment duration as a continuous variable over time.

Overall, the results show that impairing effects of benzodiazepine hypnotics on driving performance may mitigate over time following long‐term use of 3 years, although forms of neurocognitive impairment may remain. This supports the idea to take duration of treatment into account when evaluating the impact of hypnotics on individual drivers. The implication would be that classification systems that grade effects of drugs on driving should allow for differential classification of hypnotics in relation to treatment duration. The results do not support differential grading for benzodiazepine anxiolytics.

## CONFLICT OF INTEREST

J. C. Verster has received grants from Janssen, Nutricia, Red Bull, Sequential, and Takeda and acted as a consultant/advisor for 82Labs, Canadian Beverage Association, Centraal Bureau Drogisterij bedrijven, Clinilabs, Coleman Frost, Danone, Deenox, Eisai, Janssen, Jazz, Purdue, Red Bull, Sanofi‐Aventis, Sen‐Jam Pharmaceutical, Sepracor, Takeda, Transcept, Trimbos Institute, Vital Bevrages, and ZBiotics. A. Vermeeren and J. G. Ramaekers have received funding over the last 4 years from pharmaceutical companies (Eisai, Jazz, Merck, and Transcept).
